# Occurrence of Intestinal Parasites and Its Impact on Growth Performance and Carcass Traits of Pigs Raised Under Near-Organic Conditions

**DOI:** 10.3389/fvets.2022.911561

**Published:** 2022-05-23

**Authors:** Yuzhi Z. Li, Alexander D. Hernandez, Sara Major, Rick Carr

**Affiliations:** ^1^West Central Research and Outreach Center, University of Minnesota, Morris, MN, United States; ^2^Department of Biology, Kutztown University of Pennsylvania, Kutztown, PA, United States; ^3^Rodale Institute, Kutztown, PA, United States

**Keywords:** parasites, pigs, growth performance, carcass traits, production stage, sex

## Abstract

Parasite infection is a common problem in organic pig production, which can compromise health and growth of pigs, threaten food safety of pork products, and cause economic losses to organic farmers. To develop management strategies for controlling parasites, we evaluated intestinal parasite infection in pigs at different ages and of different sexes, and investigated whether parasite infection influences growth performance and carcass traits in a cross-sectional study. Fecal samples were collected from pigs (*n* = 298) raised under near-organic standards during nursery, growing, finishing, and gestating phases for analysis of fecal egg counts (FEC) of *Ascaris suum, Trichuris suis*, and *Oesophagostomum* spp. *Ascaris suum* eggs were not detected in the feces of nursery pigs. Eggs of *Ascaris suum* were found in 45%, 74%, and 0% of fecal samples of growing pigs, finishing pigs, and gestating sows, respectively, after false-positive adjustment (*P* < 0.001). Mean FEC of *Ascaris suum* was higher in infected finishing pigs than in infected growing pigs [2,502 vs. 724 eggs per gram (epg), *P* < 0.001]. No differences in percent of *Ascaris suum* positive samples or FEC of *Ascaris suum* were detected between sexes. Growth performance and carcass traits were not different between non-infected pigs and those infected with *Ascaris suum*. All pigs (*n* = 32) examined at slaughter had white spots on the liver, and 78% harbored *Ascaris suum* worms. *Trichuris suis* eggs were not detected in any fecal samples. Eggs of *Oesophagostomum* spp. were found in 7%, 0%, 1%, and 9% of fecal samples of nursery pigs, growing pigs, finishing pigs, and gestating sows, respectively, with a maximum FEC of 40 epg in all age groups. These results indicate *Ascaris suum* was the predominant parasite infecting growing and finishing pigs in the herds studied. To control *A. suum* infection, future research should investigate the efficacy of treating pigs with organically approved anthelmintics during the growing phase of production.

## Introduction

Parasite infection is a persistent problem in organic swine production (1–3), mainly due to requirement for housing pigs in bedded barns or outdoors while restricting anthelmintic use. In the United States, the National Organic Standards (4) require housing organic pigs in bedded barns with access to outdoors throughout all production stages and do not allow application of synthetic anthelmintics to pigs from 38 days prenatal (the third trimester of gestation) to slaughter. The most common parasites in organic pig farms are gastrointestinal (helminth) parasites with life stages outside of the host, such as embryonated eggs or free-living larvae in fecal material, that are directly responsible for new infections (5). Thus, pigs on bedded floors with exposure to feces face a greater risk of infection with intestinal parasites (6, 7).

Three species of intestinal parasites are commonly observed in pigs: *Ascaris suum* (*A. suum*), *Oesophagostomum* spp. (*O*. spp.), and *Trichuris suis* (*T. suis*), with *A. suum* being dominant in growing-finishing pigs, *O*. spp. in sows, and *T. suis* in pigs and sows with access to outdoors (5). Adult worms of *A. suum* live in the small intestine and *O*. spp. and *T. suis* live in different sections of the large intestine of pigs; and infections with a single species or co-infections with multiple species can occur (7–9). Pigs infected with *A. suum* show pathological lesions, commonly known as white spots, on the liver, which are caused by *A. suum* larvae migrating to the lungs via the liver, before settling in the small intestine (10–12). Livers with white spots caused by *A. suum* infection can be rejected for human food processing and lose commercial value (13). Additionally, infection can compromise the health of pigs because parasites can damage intestinal membranes and consequently compromise the immune system of the hosts (14–16). Pigs with damaged intestinal membranes may have diarrhea and reduce digestibility of dietary nutrients, which could ultimately reduce growth performance (17–19). A meta-analysis (20) indicated that intestinal parasite infection can reduce average daily feed intake (ADFI) by 5%, average daily gain (ADG) by 31%, and feed efficiency (gain:feed) by 6% in pigs.

A recent survey in the United States across fives states indicates that parasite infection is so common that all organic pig farms surveyed were infected with at least one species of intestinal parasites (2). However, research on parasite infection in organic pigs in the United States barely exists. To develop efficient management strategies for controlling parasite infection in organic pigs, we need to know occurrence and intensity of each parasite species infection, which age group of pigs should be targeted for treatment, and how parasite infection may affect growth performance, carcass traits, and liver condemnations at slaughter. The objectives of this study were to measure parasite infection in pigs at different production stages and of different sexes, and assess whether infection influences growth performance and carcass traits in an enclosed swine herd.

## Materials and Methods

This study was conducted between 2019 and 2020 at the University of Minnesota's West Central Research and Outreach Center located in the Midwestern region (Morris, Minnesota) of the United States. The experimental protocol used in the study was reviewed and approved by the University of Minnesota Institutional Animal Care and Use Committee (IACUC#: 2106-39200A).

### Animals, Housing, and Management

Pigs housed in the swine facilities with bedded floors were used for this study. The swine facilities included a hoop barn for gestating sows, an indoor barn for lactating sows and nursery pigs, and two hoop barns for growing-finishing pigs. All barns were bedded with wheat straw and had no confinement structures, except stalls in the gestation barn that were used during feeding to control individual feed intake of gestating sows. Within each barn except the gestation barn, pigs were managed all-in/all-out (AI/AO). All pigs were managed according to the National Organic Program (4), except conventional straw for bedding, piglets being weaned one week earlier than the National Organic Program requires, and pigs and sows being denied for access to outdoors due to the facility structures.

Gestating sows (Yorkshire × Landrace) were housed in 4 pens on bedded concrete floors, and each pen accommodated 15 sows. Fifteen feeding stalls and a water fountain with two drinking spaces were present in each pen. Floor space allowance in each pen was 3.7 m^2^ per sow, excluding the area occupied by feeding stalls and the water fountain. Sows entered the gestation barn after weaning and remained until 3 to 7 days before the expected farrowing dates when the sows were moved to the farrowing barn. Sows farrowed in two batches, each consisting of 30 sows from 2 gestation pens. The second batch of sows farrowed 10 weeks after the first batch. The gestation barn was managed on a continuous basis (not AI/AO). Bedding in the gestation barn was cleaned out every 1 to 3 months according to the season, less frequently in winter to preserve the heat and more frequently in summer to reduce moisture in the barn.

The farrowing barn served as both a farrowing and a nursery barn. Eight sows were housed in each of three identical rooms (9.8 m × 11.0 m) on bedded concrete floors. Sows in each farrowing room shared two feeders with 4 feeding spaces each, and one water fountain with two drinking spaces in the communal area. Sows farrowed and nursed their piglets in individual pens during the first 10 days after farrowing. Then, farrowing pens were removed so that sows and their piglets within each room mingled in a large group until weaning. Piglets were ear-tagged for individual identification, supplemented with iron via intramuscular injection, and male piglets were castrated within 24 h after birth. No piglets were tail docked or ear-notched according to the National Organic Program (4). Piglets were weaned at 5 weeks of age by removing the sows from the farrowing rooms. After weaning, piglets remained in each farrowing room for another 3 weeks. The farrowing barn was power-washed and dried after bedding being cleaned out between groups.

At 8 weeks of age, healthy pigs (Yorkshire × Landrace × Duroc) with no visual signs of illness, lameness, or any other physical injuries were transferred to growing-finishing hoop barns. Each growing-finishing barn had two pens, and each pen (6 m × 24 m) was equipped with a round bulk feeder with 12 feeding spaces and a water fountain with 4 drinking spaces on a raised concrete platform (6 m × 4 m). The rest area of the pen was bedded with straw on pressed clay floors. Fifty pigs balanced for sex and body weight were assigned to each pen within a hoop barn. Once assigned, pigs remained in their pens until they reach slaughter weight (average 125 kg). After bedding being cleaned out between groups, the growing-finishing hoop barns remained empty for 2 to 4 weeks to dry the floors.

Room temperature was controlled by a central heating system and exhaust fans to achieve a room temperature of 20°C in the farrowing and nursery barn. Unlike the farrowing and nursery barn, the hoop barns for gestation and growing-finishing pigs were ventilated naturally through openings on the sides and ends of the barns with no mechanical ventilation, heating, or cooling systems. Thermal environment in all hoop barns was maintained by adjusting the openings of the barn and the amount of bedding provided. The depth of straw bedding in the hoop barns was maintained at 40 to 60 cm during winter months, 10 to 30 cm during summer months, and 20 to 40 cm during other months. Fresh bedding was added when a new group of sows or pigs entered the barns. Throughout the rearing period, fresh bedding was added as needed to maintain clean and dry lying areas in each room and pen, and to achieve the desired microthermal environment for sows and pigs. Diets were formulated to meet or exceed nutrient requirement of pigs at each production stage recommended by the National Research Council (21). Except sows that were limited fed to achieve desired body condition during gestation, pigs and sows were provided feed *ad libitum* and water all the time. Pigs from birth to slaughter and sows from the 3^rd^ trimester of gestation (38 days prior to the expected farrowing date) to weaning were fed organically approved feed and were not treated with any antibiotics or anthelminthics. Prior to the 3^rd^ trimester of gestation, sows were treated with anthelmintics (Safe-Guard 0.9% Swine Dewormer, Zoetis Inc, with Fenbendazole as the active ingredient) by top-dressing 100 mg/kg body weight for 3 consecutive days.

### Experimental Design and Data Collection

A cross-sectional study was conducted involving pigs at four production stages: gestating sows (*n* = 45) prior to deworming, nursery pigs (*n* = 29) at 6 weeks of age, growing pigs (*n* = 91) at 15 weeks of age, and finishing pigs (*n* = 133) at 23 weeks of age. All fecal samples (*n* = 298) were collected over 8 days between 2019 and 2020. In 2019, 104 fecal samples were collected from gestating sows (*n* = 45) in 4 pens, nursery pigs (*n* = 29) in 2 rooms, and growing pigs (*n* = 30) in 2 pens over 3 days in June, September, and November, respectively. In 2020, 194 fecal samples from growing pigs (*n* = 61) in 4 pens and finishing pigs (*n* = 133) in 6 pens were collected over 5 days in January, June, August, September, and October, respectively. Among the finishing pigs that were fecal sampled, 103 pigs (25 to 27 pigs from each pen) in 4 pens were identified individually and recorded for growth performance and carcass traits (see *Section Growth performance, carcass traits, liver white spots, and A. suum worm presence*).

#### Fecal Sampling

Fecal samples were collected either during or immediately after pigs and sows defecated using the method described by Katakam et al. (1). Pigs were selected for fecal sampling based on their defecation during the time of sampling. For all pigs and most dry sows, samples were collected either before the feces was dropped on the floor or from the top of the feces that have just dropped on the floor. For 6 sows that did not defecate during the sampling periods, fecal samples were collected from the rectum. To avoid fecal samples being collected repeatedly from the same pig at a sampling time point, pigs were marked with a crayon on their back after their fecal samples were collected. Each sample (10 to 30 g/sample) was placed in a plastic zip-lock bag and stored on ice in a cooler immediately after sampling. Then the samples were stored in a refrigerator at 4°C for 6 to 72 h (median = 24 h) before being shipped to Kutztown, Pennsylvania. All fecal samples were shipped overnight on cool pads to Kutztown University for analysis of fecal egg counts (FEC) of each parasite species.

#### Lab Analysis for Parasite Fecal Egg Counts

Fecal samples were processed and analyzed within a few days (2 to 10 days, median = 4 days) of sampling. Fecal egg counts of *A. suum, O*. spp., and *T. suis* were determined according to the concentration McMaster technique described by Roepstorff and Nansen (22). Saturated NaCl-glucose solution (50 g NaCl, 75 g glucose monohydrate, and 131 g water) was used as a flotation fluid and the detection limit was 20 egg per gram (epg) of fecal sample for all three species of parasites. Fecal samples were analyzed to determine positive samples, egg abundance, and intensity for each parasite species. Positive samples were the number of fecal samples detected with parasite eggs as percent of total fecal samples analyzed. Egg abundance was the average FEC of all fecal samples collected. Egg intensity was the average FEC of fecal samples that were detected with parasite eggs (22). False-positive adjustment was performed for evaluation of *A. suum* infection, using FEC of 200 epg as the criteria according to Boes et al. (23) and Katakam et al. (1). Fecal samples with FEC of *A. suum* < 200 epg were considered false-positive of *A. suum* infection.

#### Growth Performance, Carcass Traits, Liver White Spots, and *A. suum* Worm Presence

For finishing pigs (*n* = 103) that were individually identified for fecal sampling, sex, growth performance and carcass traits were recorded for each pig. Pigs were weighed individually at 8 weeks of age when entering the hoop barn, every 4 weeks thereafter, and at 23 weeks of age prior to slaughter. Average daily gain was calculated for each pig from weight changes and days between each weighing. At 23 weeks of age, all pigs, except two pigs that did not meet requirements for slaughter and 32 pigs that were used for evaluation of liver white spots and *A. suum* worm presence, were slaughtered at a commercial meat processing plant where hot carcass weight and backfat thickness at the last rib were recorded for each pig. Dressing percentage was calculated for each pig based on carcass weight and live weight before slaughter of each pig using the equation: dressing (%) = (hot carcass weight/live weight) × 100%.

A subset of individually identified pigs (*n* = 32, all gilts) was examined for liver white spots caused by migration of *A. suum* larvae and for presence of *A. suum* worms in the small intestine when pigs were slaughtered at the Meat Lab of the University of Minnesota at 24 weeks of age [average weight = 128 kg ± 8.7 (SD)]. Liver white spots were classified arbitrarily into two categories based on the number: many (more than 10 white spots on a liver) and few (10 or fewer than 10 white spots on a liver). *Ascaris suum* worms in the small intestines were recorded as presence or absence. Meanwhile, hot carcass weight and backfat thickness at the last rib were recorded for each pig as in the commercial processing plant.

### Data Analysis

All data were analyzed using SAS software (Version 9.4; SAS Institute Inc., Cary, NC) and tested for normal distribution using the Univariate Procedure. Data for percent of positive samples were analyzed using the Frequency Procedure with Chi-square (χ^2^) test. Because data for FEC (both raw and logarithm transformed) were not normally distributed, non-parametric Friedman rank test with Cochran-Mantel-Haenszel (CMH) test based on rank scores was used to detect differences among production stages and between sexes with pen serving as a stratifying variable. Due to sparse data (majority of the data with 0 epg of FEC), sampling date was not considered in any statistical model. For finishing pigs that were individually identified, *A. suum* was the only parasite species detected in fecal samples. As a result, *A. suum* infection on growth performance (initial and final weight, ADG) and carcass traits (hot carcass weight, backfat thickness, and dressing percentage) was analyzed. Pigs were categorized as infected or non-infected based on FEC of *A. suum*. Pigs with *A. suum* FEC equal to or > 200 epg were considered infected, and pigs with *A. suum* FEC < 200 epg were defined as non-infected (23). Data for growth performance and carcass were normally distributed and analyzed using the Mixed procedure. In the statistical model, the effect of *A. suum* infection on growth performance, except for ADG, and carcass traits was analyzed with infection as the fixed effect and pen as a random effect. Data for ADG were analyzed using the Mixed procedure with repeated measures in time, and the statistical model included infection, week, and their interaction as fixed effects and pen as a random effect. In both models, infection nested within pen was used as the experimental unit and differences between least square means were tested using Tukey's test. Initial weight was used as a covariate for data analysis of final weight, hot carcass weight and ADG, final weight as a covariate for dressing percentage, and hot carcass as a covariate for backfat thickness. To analyze correlations between *A. suum* infection and pig performance, correlations of FEC with initial body weight, final body weight, ADG, carcass weight, dressing percentage, and backfat thickness at the last rib were estimated using the Spearman rank correlation procedure. Furthermore, data for the number of liver white spots and presence of *A. suum* worm in the small intestine were analyzed using the Frequency and Logistic procedures. The likelihood of *A. suum* presence associated with liver white spots was tested using the Wald Chi-square (χ^2^) for odds ratios with 95% confidence intervals. All tests were two-tailed tests. Differences were considered significant when *P* < 0.05.

## Results

### Parasite Infection and Fecal Egg Counts

Percent of positive samples and FEC (abundance and intensity) of three species of parasites in four production stages are listed in Table 1. *Ascaris suum* eggs were not detected in the feces of nursery pigs. Percent of *A. suum* positive samples was the highest in finishing pigs (91.7%), lowest in gestating sows (6.7%), and intermediate in growing pigs (75.8%; *P* < 0.001) without false-positive adjustment. After false-positive adjustment, percent of *A. suum* positive samples remains higher in finishing pigs than in growing pigs (74.4% vs. 45.1%; *P* < 0.001), and dropped to 0% in gestating sows. Mean FEC (both abundance and intensity) of *A. suum* were higher (both *P* < 0.001) in finishing pigs than in growing pigs. Confidence limits for mean FEC and range for the inner quartile (between 25^th^ and 75^th^ percentile) were wider for finishing pigs than for growing pigs, suggesting large variation in *A. suum* FEC among individual finishing pigs than among growing pigs.

**Table 1:** Positive samples and fecal egg counts, measured in eggs per gram (epg), of intestinal parasites in pigs at different ages.

	**Age of pigs**					
**Item**	**Nursery^**a**^** **(6-wk old)**	**Growers** **(15-wk old)**	**Finishers (23-wk old)**	**Dry sows** **(Parities 1–8)**	**(χ^2^)^**b**^**	***P*–value^**c**^**
Number of pigs sampled	29	91	133	45		
***Ascaris suum***						
**Positive samples (%)**						
Without false-positive adjustment^d^	0	75.8	91.7	6.7	13.2	<0.001
With false-positive adjustment^d^	0	45.1	74.4	0	19.5	<0.001
**Fecal egg count (epg)**						
**Abundance** ^**e**^						
Mean	0	354	1,932	3	37.7	<0.001
Lower–Upper CL^f^	-	248-459	1,480-2,383	-	-	-
Quartile range^g^	-	540	2,440	-	-	-
**Intensity** ^**h**^						
Without false-positive adjustment^d^						
Mean	0	467	2,106	47	26.5	<0.001
Lower–Upper CL^f^	-	338-595	1,626-2,586	-	-	-
Quartile range^g^	-	560	2,540	-	-	-
With false-positive adjustment^d^						
Mean	0	724	2,502	0	15.6	<0.001
Lower–Upper CL^f^	-	552-895	1,960-3,045	-	-	-
Quartile range^g^	-	400	2,500	-	-	-
***Oesophagostomum spp***.						
Positive samples (%)	6.9	0	0.8	8.9	14.3	0.01
***Fecal egg count (epg)***						
Mean abundance^e^	1.4	0	0.2	2.2	-	-
Maximum	20	0	20	40	-	-
Mean intensity^h^	20	0	20	25	-	-
***Trichuris suis***						
Positive samples (%)	0	0	0	0	-	-
Fecal egg count (epg)	0	0	0	0	-	-

a * Three days after weaning*.

b *Friedman's Chi-square (df = 1) was used for all variables except for A. suum positive samples without false-positive corrections and O. spp. positive samples where Chi-square (df = 3) was used*.

c *Pen was used as a stratifying variable with Cochran-Mantel-Haenszel (CMH) tests based on rank scores between 14 and 23 weeks of age*.

d *Fecal egg counts < 200 epg (egg per gram of sample) was considered as false-positive (5, 23)*.

e *Mean fecal egg counts for all samples, including samples without any parasite egg detected*.

f *Lower and Upper 95% confidence limits (CL) for the mean*.

g *The inner quartile range between 75^th^ percentile and 25^th^ percentile of fecal egg counts*.

h *Mean fecal egg counts for samples with parasite eggs detected (>= 20 epg of the detecting limit)*.

*Oesophagostomum* spp. eggs were not detected in the feces of growing pigs (Table 1). Percent of *O*. spp. positive samples was higher in nursery pigs (7%) and gestating sows (9%) than in finishing pigs (1%; *P* < 0.01), with the maximum FEC of 40 epg across all age groups. Cautions should be taken when interpreting the differences in *O*. spp. infection among production stages because most fecal samples had 0 epg of *O*. spp. *Trichuris suis* eggs were not detected in any fecal samples in this study.

Among the finishing pigs that were individually identified, 58 were barrows and 45 were gilts (Table 2). There were no differences in percentage of *A. suum* positive samples between sexes, regardless of false-positive adjustment (both *P* > 0.37). Likewise, no difference in *A. suum* FEC (abundance or intensity) was detected between sexes (all *P* > 0.41).

**Table 2:** Positive samples and fecal egg counts, measured in eggs per gram (epg), of *Ascaris suum* in barrows vs gilts at 23 weeks of age.

**Item**	**Barrows**	**Gilts**	**(χ^2^)^**a**^**	***P*–value^**b**^**
Number of pigs sampled	58	45		
**Positive samples (%)**				
Without false-positive adjustment^c^	93.1	97.8	0.82	0.37
With false-positive adjustment^c^	81.0	75.6	0.07	0.79
**Fecal egg count (epg)**				
**Abundance** ^**d**^				
Mean	2,056	2,057	0.19	0.66
Lower–Upper CL^e^	1,398–2,714	1,144–2,971	-	-
Quartile range^f^	2,560	2,680	-	-
**Intensity** ^**g**^				
Without false-positive adjustment^c^				
Mean	2,208	2,104	0.01	0.91
Lower–Upper CL^e^	1,519–2,898	1,174–3,034	-	-
Quartile range^f^	2,900	2,680	-	-
With false-positive adjustment^c^				
Mean	2,469	2,556	0.61	0.44
Lower–Upper CL^e^	1724–3,213	1,468–3,644	-	-
Quartile range^f^	3,420	2,120	-	-

a * Friedman's Chi-square (df = 1)*.

b *Pen was used as a stratifying variable with Cochran-Mantel-Haenszel (CMH) tests based on rank scores*.

c *Fecal egg counts < 200 epg (egg per gram of sample) was considered as false-positive (5, 23)*.

d *Mean fecal egg counts for all samples, including samples without any parasite egg detected*.

e *Lower and Upper 95% confidence limits (CL) for the mean*.

f *The inner quartile range between 75^th^ percentile and 25^th^ percentile of fecal egg counts*.

g *Mean fecal egg counts for samples with parasite eggs detected (>=20 epg of the detecting limit)*.

### Growth Performance and Carcass Traits

Twenty-two pigs (21%) were categorized as non-infected and 81 pigs (79%) were categorized as infected with *A. suum* (Table 3). No difference in initial or final body weight, ADG, hot carcass weight, dressing percentage, or backfat thickness at the last rib was detected between pigs that were infected and not infected with *A. suum*. Likewise, Spearman rank correlation analysis did not detect correlations of *A. suum* FEC with growth performance or carcass traits (Table 4). Two pigs were not sold for slaughter, due to mobility problems or lighter (91 kg) than the minimum slaughter weight (100 kg) required by the processing plant. Both pigs were infected with *A. suum*, with FEC of 1,060 and 2,360 epg for the pigs with mobility problems and lightweight, respectively.

**Table 3:** Growth performance and carcass traits of pigs infected vs non-infected with *Ascaris suum*.

**Item**	**Non-infected**	**Infected^**a**^**	**PooledSE^**b**^**	***P*-value**
***Ascaris suum*** **infection**^**a**^				
Number of pigs	22	81		
Body weight (kg)
Initial^c^	17.3	16.9	0.83	0.68
Final^d^, ^e^	124.9	126.6	2.68	0.57
Average daily gain (kg)	0.949	0.953	0.0178	0.87
Hot carcass weight^e^ (kg)	91.7	92.7	2.08	0.67
Dressing^f^ (%)	73.6	73.3	0.46	0.63
Backfat thickness^g^ (mm)	21.1	21.6	0.76	0.57

a * Infection was defined as pigs with fecal egg counts greater or equal to 200 eggs per gram of fecal samples*.

b *Greater SE was used for the pooled SE*.

c *Live weight when pigs were 8 weeks of age*.

d *Live weight when pigs were 23 weeks of age, prior to marketing*.

e *Initial weight was used as a co-variate*.

f *Backfat thickness was measured at the last rib, and final weight was used as a co-variate*.

g *Hot carcass weight was used as a co-variate*.

**Table 4:** Coefficient (r) of Spearman rank correlation of *Ascaris suum* fecal egg count with growth performance and carcass traits of infected finishing pigs^a^ at 23 weeks of age.

**Item**	***n***	**r**	***P*–value^**b**^**
Initial weight	81	−0.09	0.44
Final weight	80	0.08	0.50
Average daily gain	80	0.08	0.46
Carcass weight	79	0.02	0.84
Dressing percentage	79	−0.19	0.10
Backfat thickness	79	0.03	0.80

^a^*Pigs with fecal egg counts greater or equal to 200 eggs per gram of fecal samples*.

b *P-value for Spearman coefficient*.

For pigs (*n* = 32) that were examined for liver white spots and *A. suum* worm presence, 100% of the pigs had white spots on the liver, with 78% (25 out of 32 pigs) having many (more than 10) white spots on each liver. Likewise, 78% of these pigs hosted *A. suum* worms. Pigs with many white spots on the liver were more likely to harbor *A. suum* worms in the small intestine (odds ratio = 144, lower 95% confidence limit = 7.8; Wald χ^2^= 11.2, df=1; *P* < 0.001) compared to pigs with fewer white spots on the liver.

## Discussion

### Parasite Infection at Different Production Stages

Parasite infection in pigs at different production stages is influenced by both the immunogenic property of the parasite and management practices to control parasites. Generally, in organic swine production systems where pigs are raised on bedded floors and anthelmintic drugs are prohibited, *A. suum* infection is expected to be higher in pigs (nursery, growing and finishing pigs) than in breeding sows due to the high immunogenicity of *A. suum* (1, 3, 5). On the other hand, *O*. spp. infection is expected to be higher in sows than in pigs due to the low immunogenicity of the parasite (5, 7). In conventional production systems where pigs are raised in confinement barns on slatted floors to separate pigs from feces and can be treated with anthelmintic drugs when needed, *A. suum* infection does not occur until pigs reach the growing or finishing phase, and *O*. spp. remains at a very low level until pigs reach the breeding phase (5, 24). In the current study, we observed distinct patterns of intestinal parasite infections in pigs at different production stages. For instance, *A suum* infection was detected in growing and finishing pigs, but not detected in newly weaned nursery pigs or gestating sows after false-positive adjustment. On the contrary, *O*. spp. infection was detected in nursery pigs and gestating sows, but not detected in growing pigs. *Trichuris suis* was not detected in pigs of any production stages in this study, possibly because all pigs were housed in barns without access to outdoor environments (25, 26). These patterns of intestinal parasite infection in pigs at different production stages in the current study are more similar to the patterns observed in conventional production systems than the patterns in organic production systems, possibly due to housing, management, and the parasite control protocol applied to the swine herds studied.

Application of the anthelmintic drug (fenbendazole) by the 3^rd^ trimester of gestation could be a main reason for the absence of *A. suum* and *T. suis* infection in gestating sows in the current study. Fenbendazole can effectively protect sows from infections of most swine parasite species, with 100% protection against *A. suum*, and 94% to 100% protection against *T. suis* (27, 28). Additionally, washing and drying the farrowing barn could have removed and reduced the survival of parasite eggs, prevented parasite transmission from previous farrowing groups (24), and ultimately reduced parasite pressure in the sow herds in this study. Furthermore, sows housed in the bedded barns possibly could have developed immunity against intestinal parasites, especially *A. suum* and *T. suis*. which can stimulate strong immune reactions in pigs (5). Compared to *A. suum* and *T. suis, O*. spp. does not stimulate strong immune responses in pigs, which could contribute infection of *O*. spp. observed in gestating sows in the current study. However, percent of *O*. spp. positive samples (9%) in sows in the current study was much lower than reported for organic sows (e.g., 50% reported by 5; 20% reported by 6) and sows across different housing systems (e.g., 52% reported by 7). Additionally, *O*. spp. egg intensity in sows in the current study was barely detectable. The low percent of positive samples combined with low egg intensity suggests that *O*. spp. infection is not a concern for the sow herds in the current study.

Percent of positive samples and egg intensity of *A. suum* and *O*. spp. in nursery pigs followed similar patterns as in gestating sows in the current study. While *A. suum* was not detected in any fecal samples of nursery pigs, we could not confirm that nursery pigs were not infected with *A. suum* in the current study. This is because the prepatent period of *A. suum* is 6 to 8 weeks (5, 12), which is close to the age of nursery pigs in this study. Using experimental inoculation, Mejer and Roepstorff (29) and Nejsum et al. (12) observed that most pigs started to secrete *A. suum* eggs between 7 and 11 weeks of age post inoculation. Piglets born to contaminated pastures did not shed *A. suum* eggs in their feces until 9 weeks of age (29). Likewise, Lindgren et al. (3) reported that 12-week old pigs on organic farms shed significant amount (138 to 7,612 epg) of *A. suum* eggs in feces. If the nursery pigs in the current study had been infected with *A. suum*, we expect both percent of positive samples and egg intensity would be low due to the minimal shedding of *A. suum* eggs by the sows that farrowed and nursed the pigs. On the other hand, percent of *O*. spp. positive samples and egg intensity of *O*. spp. in nursery pigs in this study were much lower than in organic nursery pigs reported previously (6, 24). The low percent of positive samples (7%) combined with barely detected FEC (20 epg) renders *O*. spp. not being a concern for nursery pigs studied. Interestingly, percent of *O*. spp. positive samples in growing (0% prevalence) and finishing (<1% prevalence) pigs was even lower than in nursery pigs in the current study. These data suggest that growing and finishing pigs in the current study barely harbored *O*. spp. worms.

Adjustment for false-positive infection of *A. suum* was used in this study to exclude pigs that were not infected but only passed *A. suum* eggs through their digestive tracts. Boes et al. (23) and Katakam et al. (1) reported that pigs with FEC < 200 epg were not likely to harbor *A. suum* worms, instead they only passed parasite eggs through the digestive tracts due to coprophagy. Coprophagy is more frequently observed in pigs that are housed indoors with high stocking density compared to pigs on pastures or housed indoors with low stocking density (23). False-positive *A. suum* samples can be as high as 30% in growing-finishing pigs, and 15% in gestating sows (1). So, adjustment for false-positive infection of *A. suum* is recommended, especially for pigs housed indoors without outdoor access, as in the current study (1, 23). After adjustment for false-positive infection, percent of *A. suum* positive samples was reduced by 30% in growing pigs and 18% in finishing pigs in the current study, which were consistent with results reported by Katakam et al. (1).

Results of the current study suggest that *A. suum* infection was a major concern for growing and finishing pigs in the swine herds studied. We observed the same trend on commercial organic pig farms where *A. suum* infection was most common in growing-finishing pigs compared to nursery pigs and gestating sows (2). Infection of *A. suum* has also been confirmed in organic fattening pigs in European countries (30). In fact, *A. suum* is the toughest parasite to control in swine due to the large amount of eggs that each female *A. suum* worm sheds daily, and because the thick shell of *A. suum* eggs that can allow the eggs to remain viable for four to ten years (5). We suspect that most of the pigs in the current study had been infected with *A. suum* in the growing-finishing hoop barns because the barns with clay floors could not be washed or disinfected between groups. Thoroughly cleaning, washing, and drying floors between groups of pigs could have reduced *A. suum* infection in growing-finishing pigs in the current study. Pettersson et al. (24) attributed the reduction in parasite prevalence in Swedish pigs over the last 30 years to improved hygiene practices. To control *A. suum* in conventional swine production systems where there is no restriction on anthelmintic treatments, it is recommended to treat sows before moving into the farrowing barn, and to treat pigs at weaning and once or twice during the growing-finishing phase (5). In organic swine production systems where synthetic anthelmintics are prohibited for market pigs, *A. suum* could be managed by limited application of anthelmintics that are approved for organic production. Currently, some organically approved anthelmintics are commercially available and recommended to be used for treating pigs at weaning in the Midwestern region of the United States. However, results of the current study and previous work (28) suggest that treating pigs during the growing phase may be more effective to minimize transmission of *A. suum* infection than treating pigs at weaning. One reason for doing this is that pigs do not shed *A. suum* eggs at weaning as demonstrated in the current study and previous work (28). Instead, deworming pigs with organically approved anthelmintics during the growing phase can reduce the risk of *A. suum* transmission because about 30% to 50% of growing pigs shed *A. suum* eggs as observed in the current study and previously (6). Additionally, deworming during the growing period might prevent pigs from shedding *A. suum* eggs after anthelminthic treatment until slaughter due to the prepatent period length (5, 12). The efficacy of treating pigs during the growing phase of production with organically approved anthelmintics needs to be investigated in future research.

### Parasite Infection Between Sexes

We did not detect a difference in *A. suum* infection between barrows and gilts in the current study. This is consistent with results reported by Amadi et al. (31) and Aiyedun and Oludairo (32) who did not detect any difference in *A. suum* infection between sexes of pigs. Sex differences in parasite infection have been observed in vertebrates, including lab animals, field animals (field mice and voles) and pastured cattle, with males having higher infection prevalence and egg intensity of parasites than females due to the negative effect of testosterone and positive effect of progesterone against parasite infections (33). However, such difference in parasite infection between sexes has not been observed in pigs. On the contrary, Tamboura et al. (34) and Sah (35) reported that infection prevalence of *A. suum* was higher in gilts than in barrows, probably due to difference in coprophagy between sexes. Likewise, Baskota and Shrestha (36) noted that gilts showed more clinical symptoms of *A. suum* infection than barrows. Male pigs in the current study, as in commercial swine production in the United States, were castrated at birth, and castrated males (barrows) produce limited testosterone. Therefore, we do not expect any difference in testosterone concentrations between barrows and gilts. Additionally, finishing pigs in the current study did not reach their reproductive age, so progesterone effects of gilts on parasite infection are not expected.

### Effects of Parasite Infection on Growth Performance and Carcass Traits

Parasite infection can potentially result in economic losses as indicated by reduced ADFI, ADG, and feed efficiency in previous work (18, 20, 37). Yang et al. (38, 39) reported that parasite infection can cause anorexia, resulting in reduced feed intake in pigs. Reduced feed intake is one of the major reasons for reduced growth rate. Additionally, parasites can negatively affect host's nutrient absorption by causing lesions on the intestinal membranes or microvillus atrophy (14–16). As a result, the negative effect of parasite infection on ADG can be escalated when pigs are fed diets that do not meet their nutrient requirements (40, 41). Furthermore, parasite infection evokes immune response of the host and activates macrophages to produce pro-inflammatory cytokines. The cytokines can reduce growth hormone secretion in the central nervous system (42), resulting in reduced growth in parasite infected pigs. However, in the current study, we did not detect differences in growth performance or carcass traits between pigs infected and non-infected with *A. suum*. We could not evaluate the difference in feed intake between infected and non-infected pigs in the current study because both infected and non-infected pigs were housed in the same pen (50 pigs/pen) and fed as a group. However, we speculate that both infected and non-infected pigs consumed enough feed to meet their nutrient requirements because pigs in the current study were fed *ad libitum* and all diets were formulated to meet or exceed nutrient requirements of pigs according to the NRC (21) recommendations. Consequently, there was no difference in growth performance or carcass traits between infected and non-infected pigs in the current study. Our results are consistent with results of Martinez-Perez et al. (43) who reported that *A. suum* infection was not correlated with ADG in pigs. On the other hand, Bernardo et al. (44) reported that the number of *A. suum* worms in the small intestine did not affect ADG, but the life-time burden of *A. suum*, measured by FEC and the duration of infection, was negatively associated with ADG. Additionally, Knecht et al. (17), Jankowska-Makosa and Knecht (18) reported that pigs infected with both *A. suum* and *O*. spp. reduced ADG, dressing and carcass lean in pigs. Because we did not observe *O*. spp. infection in growing pigs in the current study, our results are not comparable to results of Knecht et al. (17).

Another potential economic loss caused by *A. suum* infection is liver condemnation (43). In the current study, we observed white spots on all livers examined, suggesting that all pigs may have infected with *A. suum* during the rearing phase (10, 11, 45). However, not all these pigs harbored *A. suum* worms at slaughter. We observed pigs that had more than 10 white spots on the liver were likely to harbor *A. suum* worms at slaughter. In other words, livers from pigs hosting *A. suum* worms are more likely to be excluded from the food processing chain, resulting in economic losses to organic pork farmers. Several researchers reported evidence that pigs may develop a “pre-hepatic barrier” after continuous exposure to *A. suum* and presence of *A. suum* worms in the small intestine (45–47). The pre-hepatic barrier is considered to regulate *A. suum* worm population in the small intestine of the host by preventing ingested larvae migrating to the liver (45–47). However, some later studies (12, 29) demonstrated that the pre-hepatic barrier did not completely prevent ingested larvae from migrating to the liver. In fact, Nejsum et al. (12) reported that the presence of *A. suum* worms in the intestine does not affect larvae migrating to the lungs, the liver, or the intestine, but affects whether larvae could survive in the small intestine and grow to adult worms. This may explain results from the current study that pigs that hosted *A. suum* worms had more white spots on the liver, possibly because these pigs could have been continuously infected with *A. suum*, resulting in larvae continuously migrated to the liver. In the current study, 78% of focal pigs examined for *A suum* worm presence in the small intestine hosted *A. suum* worms, which was consistent with percent of *A. suum* positive samples (78.6% after false-positive adjustment) in the 103 pigs from which the focal pigs were derived. This further suggests that false-positive adjustment was essential for evaluation of *A. suum* infection using FEC in enclosed herds as in the current study.

## Conclusion

In the enclosed swine herds studied, intestinal parasite infection was not a concern for gestating sows and nursery pigs because sows were treated with anthelmintic drugs by the 3^rd^ trimester of gestation and farrowed in a clean farrowing barn. Growing and finishing pigs were heavily infected with *A. suum* in hoop barns on bedded clay floors which may have been contaminated with *A. suum* over the years of operation. However, *A. suum* infection did not affect growth performance or carcass traits, and there was no difference in *A. suum* infection between sexes in the current study. Pigs that harbored *A. suum* worms bore many (more than 10) white spots on the liver, which can result in rejection of the liver for use as human food. Management strategies for controlling *A. suum* infection in organic pigs, including application of organically approved anthelmintics during the growing phase of production, need to be investigated in the future.

## Data Availability Statement

The raw data supporting the conclusions of this article will be made available by the authors, without undue reservation.

## Ethics Statement

The animal study was reviewed and approved by University of Minnesota Institutional Animal Care and Use Committee.

## Author Contributions

YL designed and conducted the animal trial, analyzed the data, and prepared the manuscript. AH and SM conducted lab analysis and corrected the manuscript. RC reviewed and corrected the manuscript. All authors contributed to and approved the submitted version.

## Funding

This work was supported by the Organic Transition Program (Award#: 2018-51106-28772) from the USDA National Institute of Food and Agriculture.

## Conflict of Interest

The authors declare that the research was conducted in the absence of any commercial or financial relationships that could be construed as a potential conflict of interest.

## Publisher's Note

All claims expressed in this article are solely those of the authors and do not necessarily represent those of their affiliated organizations, or those of the publisher, the editors and the reviewers. Any product that may be evaluated in this article, or claim that may be made by its manufacturer, is not guaranteed or endorsed by the publisher.
